# Rifabutin Is Active against Mycobacterium abscessus Complex

**DOI:** 10.1128/AAC.00155-17

**Published:** 2017-05-24

**Authors:** Dinah Binte Aziz, Jian Liang Low, Mu-Lu Wu, Martin Gengenbacher, Jeanette W. P. Teo, Véronique Dartois, Thomas Dick

**Affiliations:** a Department of Microbiology and Immunology, Yong Loo Lin School of Medicine, National University of Singapore, Singapore; b Department of Laboratory Medicine, National University Hospital, Singapore; c Public Health Research Institute, New Jersey Medical School, Rutgers, The State University of New Jersey, Newark, New Jersey, USA

**Keywords:** Mycobacterium abscessus, NTM, rifabutin, repurposing

## Abstract

Lung infections caused by Mycobacterium abscessus are emerging as a global threat to individuals with cystic fibrosis and to other patient groups. Recent evidence for human-to-human transmission worsens the situation. M. abscessus is an intrinsically multidrug-resistant pathogen showing resistance to even standard antituberculosis drugs, such as rifampin. Here, our objective was to identify existing drugs that may be employed for the treatment of M. abscessus lung disease. A collection of more than 2,700 approved drugs was screened at a single-point concentration against an M. abscessus clinical isolate. Hits were confirmed with fresh solids in dose-response experiments. For the most attractive hit, growth inhibition and bactericidal activities against reference strains of the three M. abscessus subspecies and a collection of clinical isolates were determined. Surprisingly, the rifampin derivative rifabutin had MICs of 3 ± 2 μM (3 μg/ml) against the screening strain, the reference strains M. abscessus subsp. abscessus ATCC 19977, M. abscessus subsp. bolletii CCUG 50184-T, and M. abscessus subsp. massiliense CCUG 48898-T, as well as against a collection of clinical isolates. Furthermore, rifabutin was active against clarithromycin-resistant strains. In conclusion, rifabutin, in contrast to rifampin, is active against the Mycobacterium abscessus complex bacteria *in vitro* and may be considered for treatment of M. abscessus lung disease.

## INTRODUCTION

Infections caused by nontuberculous mycobacteria (NTM) are being recognized as real and rising threats (1–3). Mycobacterium abscessus is the most frequently encountered rapid-growing NTM implicated in human disease (2, 4, 5). The bacterium most commonly causes pulmonary infections in patients with immune deficiencies or with a predisposed lung condition, such as cystic fibrosis or bronchiectasis (1, 6–8). Until recently, it was believed that M. abscessus infections were exclusively acquired by exposure to contaminated soil or water. However, a study by Bryant et al. demonstrated human-to-human transmission in cystic fibrosis patients (5, 9).

The taxonomy of M. abscessus has been highly dynamic (10–12). Recent large-scale genome analyses demonstrated separation of M. abscessus into three divergent subspecies—M. abscessus subsp. abscessus, M. abscessus subsp. bolletii, and M. abscessus subsp. massiliense (9, 12, 13)—challenging an earlier classification of M. abscessus into only two subspecies (10).

M. abscessus infection is notoriously difficult to treat, as the bacterium shows a high level of intrinsic drug resistance to many antibiotics, including those used for treatment of Mycobacterium tuberculosis infections, such as rifampin (14, 15). Standard of care calls for 12 months of negative sputum cultures while on therapy (1, 16), which can result in several years of treatment with a minimum of three antibiotics. Because of the poorly performing chemotherapy, surgical resection of M. abscessus lung lesions is recommended when possible (1, 5). Macrolides, specifically clarithromycin, are part of multidrug regimens which include parenteral drugs, such as amikacin, as well as the beta-lactams imipenem and cefoxitin (1, 2, 7, 16). The already poorly performing treatments for M. abscessus infections are further complicated by the widespread occurrence of strains displaying inducible clarithromycin resistance (17, 18). Inducible clarithromycin resistance is mediated by the *erm*(41) gene, which encodes a methyl transferase that modifies the ribosomal binding site of clarithromycin (19–21). Although *erm*(41) sequences appear to be present in all M. abscessus subspecies, inducible macrolide resistance appears to occur mainly in M. abscessus subsp. abscessus and M. abscessus subsp. bolletii but not in M. abscessus subsp. massiliense, which carries deletions in the coding sequence of the gene which render the enzyme nonfunctional (20, 21). Not all M. abscessus subsp. abscessus strains harbor inducible clarithromycin resistance. A subset of clinical isolates of this subspecies carrying a polymorphism within its *erm*(41) coding sequence does not express inducible clarithromycin resistance (21, 22).

Despite an urgent medical need to discover new antimycobacterials or repurpose existing drugs, there is a distinct lack of activity and progress in drug discovery for treating NTM infections (1, 11, 23–25). Here, we asked whether the repertoire of existing drugs and antibiotics may contain overlooked medicines showing anti-M. abscessus activity for rapid bench-to-bedside translation. Repurposing of drugs has proven effective in finding potential candidates for treatment of bacterial infections caused by methicillin-resistant Staphylococcus aureus, Acinetobacter baumannii, Pseudomonas aeruginosa, M. tuberculosis, and others (26–29). There is precedent for treatment of M. abscessus infection where the authors identified the nitroimidazole metronidazole as a potent antibiotic against M. abscessus (14). However, the result could not be reproduced (30).

We screened 2,720 approved drugs against a clinical isolate of M. abscessus. Surprisingly, we found that rifabutin, a derivative of the poorly active rifampin, was a potent growth inhibitor of the screening strain. *In vitro* activities of rifabutin against reference strains and clinical isolates were characterized.

## RESULTS

### Screening of approved drugs identifies rifabutin as a potent inhibitor of M. abscessus Bamboo.

**FIG 1:**
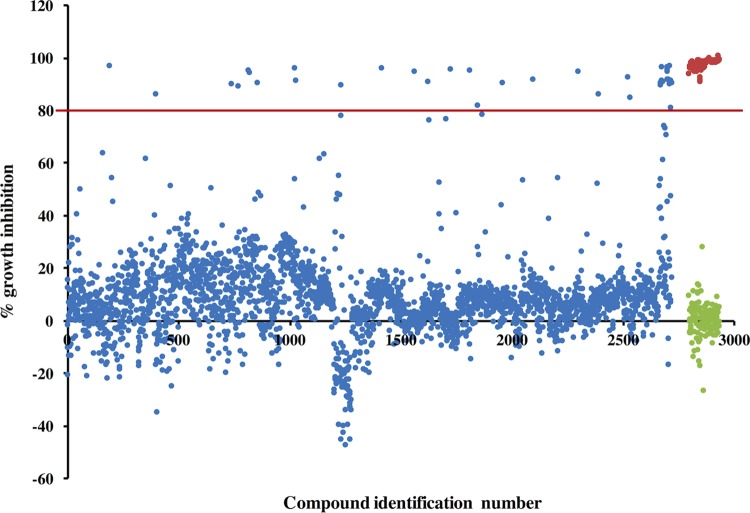
Primary screen compound–growth inhibition scatter plot. A total of 2,720 drugs at a concentration of 20 μM were screened for growth inhibition against M. abscessus Bamboo. Test drugs are shown in blue, drug-free control is in green, and clarithromycin (the positive drug control) is shown in red. The red line indicates our cutoff point of 80% growth inhibition.

To identify existing drugs that may be repurposed for the treatment of M. abscessus infections, we screened a collection of 2,720 approved drugs at 20 μM for their growth inhibition potential against the clinical isolate M. abscessus Bamboo. The screen revealed 31 primary antibiotic hits (1.1% hit rate) when a cutoff of 80% growth inhibition was applied (Fig. 1). Solids of primary hits were repurchased and tested in dose-response experiments, resulting in 17 confirmed hits (Table 1).

**TABLE 1:** Confirmed antibiotic hits active against M. abscessus Bamboo screening strain

Antibiotic class	Antibiotic	MIC_90_ (μM)
Macrolide	Azithromycin	6
	Clarithromycin	0.4
	Erythromycin	34
Aminoglycoside	Amikacin	14
	Gentamicin	9
	Kanamycin	11
Fluoroquinolone	Ciprofloxacin	8
	Gatifloxacin	5
	Levofloxacin	18
	Moxifloxacin	4
Oxazolidinone	Linezolid	36
Glycylcycline	Tigecycline	9
Ketolide	Telithromycin	4
Glycopeptide	Vancomycin	12
	Teicoplanin	17
	Ramoplanin	24
Rifamycin	Rifabutin	3

Based on literature data, expected hits included the macrolides, aminoglycosides, and fluoroquinolones (15, 31, 32), as well as newer drugs, such as the oxazolidinone linezolid, the glycylcycline tigecycline, and the ketolide telithromycin (1). Novel hits which have, to our knowledge, not been reported to be used for treatment of M. abscessus infection were the glycopeptides vancomycin, teicoplanin, and ramoplanin. However, the potencies of these glycopeptides were only modest (MIC_90_s, 12, 17, and 24 μM, respectively).

**FIG 2:**
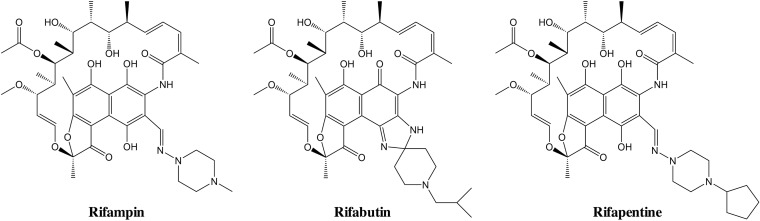
Structures of tested rifamycins.

Unexpectedly, we found that the rifamycin rifabutin had an MIC_90_ of 3 μM against the primary screening strain (Table 1). This was surprising because the structurally similar tuberculosis drug rifampin (Fig. 2) is reported to be only poorly active against M. abscessus (33) and therefore is not used in clinical practice (34). Rifabutin is successfully used for treatment of lung infections caused by M. tuberculosis, where it shows attractive pharmacokinetic properties (see Discussion). As M. abscessus causes lung infections with pathologies similar to its slow-growing relative, rifabutin may be a candidate for repurposing. Therefore, we characterized the activity of rifabutin against M. abscessus in more detail.

### Rifabutin is active against reference strains representing all three subspecies of the M. abscessus complex.

To determine whether rifabutin shows similar attractive potencies across the three subspecies of the M. abscessus complex, we measured its MICs against the reference strains M. abscessus subsp. abscessus ATCC 19977, M. abscessus subsp. bolletii CCUG 50184-T, and M. abscessus subsp. massiliense CCUG 48898-T. Rifabutin showed low MIC_90_s against all three subspecies (Table 2). These results suggest that rifabutin is active across the phylogenetically divergent M. abscessus complex. Table 2 also shows that rifampin and rifapentine displayed poor potency against the reference strains, as expected (7, 15, 35). The approximately 10-fold difference in potency of the three rifamycins is intriguing, considering the minor structural differences between the three molecules (Fig. 2).

**TABLE 2:** Potencies of rifabutin against reference strains representing subspecies of the M. abscessus complex compared to those of clarithromycin and other rifamycins^*a*^

Strain (*erm*[41] sequevar)^*b*^	MIC_90_ (μM)^*c*^				MIC_90_ (μM) after RFB pretreatment^*d*^	MBC_90_ (μM)	
	RFB	CLR	RIF	RFP	RFB	RFB	CLR
M. abscessus subsp. abscessus ATCC 19977 (T28)	3	3	37	31	4	6	12.5
M. abscessus subsp. bolletii CCUG 50184-T (T28)	4	5	>50	31	4	6	25
M. abscessus subsp. massiliense CCUG 48898-T (deletion)	1	0.4	39	13	0.7	6	>50

a In Middlebrook 7H9 broth. The experiments were repeated independently two times and mean values are shown. Standard deviations were ± 50% of the shown values. The rifabutin results shown are from drug purchased from Sigma-Aldrich. The MIC experiments were repeated with drug purchased from a different source, Adooq BioScience, and delivered identical results.

b Clarithromycin resistance gene *erm*(41) sequevars: T28 indicates inducible clarithromycin resistance; deletion of *erm*(41) indicates a nonfunctional gene and thus a clarithromycin-sensitive strain.

c RFB, rifabutin; CLR, clarithromycin; RIF, rifampin; RFP, rifapentine.

d After RFB pretreatment, prior to MIC_90_ determination, cultures were exposed to a subinhibitory concentration of rifabutin to identify possible inducible rifabutin resistance (see Materials and Methods for details).

Taken together, these results show that rifabutin retains its activity across the phylogenetically divergent M. abscessus complex and confirm that the drug is more potent than rifampin and rifapentine.

### Exposure of M. abscessus to subinhibitory concentrations of rifabutin does not trigger inducible drug resistance.

To determine whether M. abscessus subspecies may harbor any (unknown) inducible rifabutin resistance mechanisms, we pretreated cultures of the three subspecies reference strains with subinhibitory concentrations of rifabutin and measured the impact of antibiotic preexposure on their MIC_90_s. Pretreatment of bacteria with the drug did not affect the MIC_90_s, suggesting that M. abscessus does not harbor inducible rifabutin resistance mechanisms (Table 2).

### Rifabutin is bactericidal for all three subspecies of M. abscessus.

To determine whether rifabutin shows bactericidal activity against M. abscessus, cultures were treated with the drug, and the effect on viability was determined by CFU enumeration. The minimum bactericidal concentration (MBC_90_), or concentration that kills 90% of the bacteria, was approximately 2-fold the MIC_90_, i.e., rifabutin displayed similar or higher bactericidal activities compared to those of clarithromycin (Table 2).

### Rifabutin shows potent activity against clinical isolates of the M. abscessus complex.

Rifabutin showed potent growth inhibition activity against the screening strain, as well as against reference strains representing the three subspecies of M. abscessus. This suggests that most clinical M. abscessus strains may be susceptible to this rifamycin. To provide evidence for widespread susceptibility of M. abscessus to rifabutin, we tested its activity against a collection of clinical isolates covering the various subspecies of the M. abscessus complex, including clarithromycin-resistant and clarithromycin-sensitive strains. All isolates were uniformly susceptible to rifabutin, with MIC_90_ values ranging from 3 to 5 μM (Table 3). This result indicates that a large fraction of disease-causing M. abscessus strains may be susceptible to rifabutin.

**TABLE 3:** Inhibitory potency of rifabutin against clinical isolates of M. abscessus^*a*^

Isolate code	M. abscessus subspecies^*b*^	*erm41* sequevar^*c*^	Clarithromycin susceptibility	Rifabutin MIC_90_ (μM)
M9	abscessus	T28	Resistant	5
M199	abscessus	T28	Resistant	5
M337	abscessus	T28	Resistant	3
M404	abscessus	C28	Sensitive	4
M421	abscessus	T28	Resistant	3
M422	abscessus	T28	Resistant	3
M232	bolletii	T28	Resistant	3
M506	bolletii	C28	Sensitive	3
M111	massiliense	deletion	Sensitive	4

a The experiments were repeated two times independently, and mean values from those experiments are shown. Standard deviations were ±50% of the values shown.

b Subspecies were determined by sequencing *rpoB* and *hsp65*.

c The *erm*(41) sequevar was determined by sequencing the gene. For all strains, *rrl* (23S rRNA) was sequenced and found to be wild type (see Materials and Methods for details).

### Rifamycin activities shift in Mueller-Hinton medium.

All activity determinations so far were carried out in standard Middlebrook-based mycobacterial growth medium typically used in antimycobacterial drug discovery. Drug susceptibility testing for M. abscessus is carried out mostly in Mueller-Hinton medium (36, 37). To determine whether activities of rifamycins differ in the two broth types, we measured the MICs of the three rifamycins against the type strains representing the three subspecies of M. abscessus and grown in cation-adjusted Mueller-Hinton medium. Table 4 shows that rifabutin MICs showed a 2- to 3-fold increase. Similar shifts were observed for the other rifamycins. These results show that the activities of rifamycins are reduced in Mueller-Hinton medium. The relevance of this discrepancy remains to be determined (see Discussion).

## DISCUSSION

Due to its poor *in vitro* activity, the antituberculosis drug rifampin is not used in clinical practice for the treatment of lung disease caused by M. abscessus. Here, we confirm the poor activity of rifampin and report the surprising finding that its close derivative rifabutin shows attractive activity against reference strains representing the three subspecies of the M. abscessus complex and against a collection of clinical isolates. Rifabutin showed activity against widely spread clarithromycin-resistant strains, and the bactericidal activity of the drug against the three subspecies was comparable to or better than that of clarithromycin. These results suggest that rifabutin may be considered for treatment of M. abscessus infections, including lung disease. Rifabutin is orally bioavailable and is used successfully in the treatment of tuberculosis lung disease, which causes similar pathologies, defined for the most part as nodular or cavitary diseases (38). A rifamycin in general and rifabutin in particular would be a welcome addition to anti-M. abscessus drug regimens for several reasons: (i) rifamycins are active against M. tuberculosis persisters (39), and this is likely to apply to M. abscessus as well; (ii) rifabutin has a long half-life, it exhibits high intracellular penetration and a high volume of distribution, and lung/plasma concentration ratios measured in resected human lung tissue were around 6 to 7, indicating that adequate concentrations are reached at the site of infection, since steady-state plasma levels in patients receiving the standard dose of 300 mg peak around 600 to 700 ng/ml (40, 41); (iii) rifabutin is less prone to drug-drug interactions than other rifamycins due to its reduced induction of CYP3A4 (42, 43); and (iv) rifabutin is well tolerated by a large proportion of patients who develop rifampin-related adverse events (44). Despite all these potentially positive aspects, it needs to be noted that the MIC values of rifabutin of about 3 μM against strains of the M. abscessus complex are higher than the MIC values of the drug against M. tuberculosis (45, 46) and M. avium (47), i.e., the therapeutic value of this rifamycin for treating M. abscessus disease remains to be determined.

An intriguing result is the difference in activities of the three structurally related RNA polymerase inhibitors, rifabutin, rifampin, and rifapentine (Fig. 2). We are investigating whether the difference in their antibacterial activities is due to differences in intrabacterial pharmacokinetic properties of the rifamycin derivatives, i.e., differences in bacterial drug uptake, efflux, or metabolism (45, 48, 49). Understanding the mechanistic basis of the activity differences may open new avenues to inform medicinal chemistry efforts and discover more potent rifamycins for the treatment of mycobacterial infections. Interestingly, Rominski and colleagues (50) recently showed via elegant genetic studies, including heterologous expression and gene knockout studies, that *MAB_0591*, encoding a putative rifampin ADP-ribosyltransferase (48, 51–53), is a major contributor to the high level of intrinsic rifampin resistance in M. abscessus subsp. abscessus ATCC 19977 (50). It remains to be determined whether rifabutin is less metabolized by this or other putative rifampin-metabolizing enzymes, such as FAD monooxygenases (51, 54, 55), or whether the differential antibacterial activity of the rifamycins against M. abscessus is due to differences in uptake or efflux.

Why has the activity of rifabutin against M. abscessus been “overlooked” so far? A search of the literature gave few results (56). A study where 31 antimicrobials, including rifabutin, were tested against the reference strain M. abscessus subsp. abscessus ATCC 19977 revealed an MIC of 32 mg/liter for rifabutin (33), clearly higher than the value of 3 mg/liter (3 μM) found in this work. This difference may be due to the differences in assay media and conditions (Mueller-Hinton versus standard mycobacterial Middlebrook 7H9 broth) (39), as well as methods (indirect redox activity-based alamarBlue readout for growth versus direct turbidity measurement) (39). When we replaced Middlebrook 7H9 with cation-adjusted Mueller-Hinton medium in our MIC assay, leaving all other parameters (including the readout) unchanged, we observed a 2- to 3-fold shift of the Middlebrook-based MICs for all three rifamycins, confirming higher activity of rifabutin relative to those of rifampin and rifapentine and indicating a weak effect of medium on activity.

Another study by van Ingen et al. used an agar dilution method (defining MIC as the concentration that inhibits >99% of growth) to determine susceptibility of a large number of NTM species, including 82 clinical M. abscessus isolates, for a number of antibiotics, including rifabutin (47, 57). The authors deemed M. abscessus “resistant” to rifabutin based on the Clinical and Laboratory Standards Institute (CLSI) breakpoint of 2 mg/liter. However, this breakpoint was set based on data from the slow-growing Mycobacterium avium. The authors acknowledged that the selected breakpoint was not based on clinical outcome or presence of mutations in *rpoB* and may not hold true for rapid-growing mycobacteria (47, 57). The same study determined the median MIC of rifabutin for M. abscessus to be >5 mg/liter, similar to that of rifampin, suggesting that both drugs may have high MIC values (47, 57), and apparently contradicting our data. However, the authors used 5 mg/liter as the maximum concentration tested for both rifabutin and rifampin. Thus, due to the assay conditions chosen and concentration range and breakpoints selected, the authors may have missed the potency difference between the two drugs, which we detected in broth dilution dose-response experiments, where we covered a wider range of drug concentrations.

The current study has a few limitations. Susceptibility testing against a larger collection of clinical isolates need to be carried out. Furthermore, development of resistance needs to be studied, and *in vitro* and *in vivo* drug combination studies need to be conducted. Nevertheless, our results support further testing of rifabutin in preclinical pharmacokinetic/pharmacodynamic (PK/PD) models, including mouse models of infection, as well as the characterization of the drug's pharmacokinetics in patients infected with M. abscessus. Taken together, the current report indicates that rifabutin may be useful as an add-on in the treatment of chronic, largely incurable M. abscessus pulmonary disease.

## MATERIALS AND METHODS

### Compounds.

A collection of 2,662 drugs approved by the U.S. Food and Drug Administration (FDA) was provided by Vincent Smeraglia and David Kimball from Rutgers University's Office of Research and Economic Development. An in-house collection of 58 antibiotics obtained from commercial sources was included in the screen, resulting in a total of 2,720 compounds screened. The compounds from the FDA library were dissolved in 90% dimethyl sulfoxide (DMSO), and the antibiotics from the in-house library were dissolved according to the manufacturers' recommendations. For confirmation of hits identified from screening the FDA drug collection, compounds were purchased from commercial sources. Rifabutin was obtained from two independent sources, Sigma-Aldrich and Adooq BioScience, and dissolved in 90% DMSO.

### Bacterial strains and culture media.

For screen and hit confirmation, Mycobacterium abscessus Bamboo was used. M. abscessus Bamboo was isolated from the sputum of a patient with amyotrophic lateral sclerosis and bronchiectasis and was provided by Wei Chang Huang, Taichung Veterans General Hospital, Taichung, Taiwan. M. abscessus Bamboo whole-genome sequencing showed that the strain belongs to M. abscessus subsp. abscessus and harbors an inactive clarithromycin-sensitive *erm*(41) C28 sequevar (GenBank accession no. MVDX00000000) (21, 64).

For dose response and bactericidal activity determination of subspecies of the M. abscessus complex, the reference strains for the three M. abscessus subspecies were used, Mycobacterium abscessus subsp. abscessus ATCC 19977, harboring the inducible clarithromycin resistance-conferring *erm*(41) T28 sequevar (51), Mycobacterium abscessus subsp. bolletii CCUG 50184-T, harboring the inducible clarithromycin resistance-conferring *erm*(41) T28 sequevar (58), and Mycobacterium abscessus subsp. massiliense CCUG 48898-T, harboring the nonfunctional *erm*(41) deletion sequevar (13). The reference strains were purchased from the American Type Culture Collection (ATCC) and the Culture Collection University of Goteborg (CCUG), respectively.

For the dose-response studies of clinical isolates covering the M. abscessus complex, strains from the clinical microbiology laboratory at the National University Hospital in Singapore were used. The subspecies for each isolate was determined by multilocus sequencing employing the *rpoB* and *hsp65* genes (59, 60). Primer pairs 5′-GACGACATCGACCACTTCGG-3′ and 5′-GGGGTCTCGATCGGGCACAT-3′ (for *rpoB*) and 5′-ATCGCCAAGGAGATCGAGCT-3′ and 5′-AAGGTGCCGCGGATCTTGTT-3′ (for *hsp65*) were used for PCR amplification. Amplicon sequencing was performed and the gene sequences compared to those available in the GenBank database using BLASTN. Phylogenetic trees were built for each gene target using MegAlign software (DNASTAR, Madison, WI) and analyzed by bootstrap analysis with 1,000 resamplings and 111 seeds. Reference genes *hsp65* and *rpoB* were derived from whole-genome sequences for M. abscessus subsp. bolletii strain MM1513 (GenBank accession no. CP009447.1), M. abscessus subsp. massiliense strain GO 06 (CP003699.2), and M. abscessus strain FLAC054 (CP014961.1). The *erm*(41) and *rrl* genes were analyzed to determine clarithromycin resistance. Primers for *erm*(41) amplification were 5′-GACCGGGGCCTTCTTCGTGAT-3′ and 5′-GACTTCCCCGCACCGATTCC-3′ (20). The *rrl* gene was amplified using primers 5′-GTAGCGAAATTCCTTGTCGG-3′ and 5′-TTCCCGCTTAGATGCTTTCAG-3′ (21). For *erm*(41), the full-length gene sequence of 673 bp was examined for T28C polymorphism and deletions. For the *rrl* gene, the nucleotide region spanning nucleotides 2058 to 2059 was examined. Mutations at 2058 to 2059 are known to be responsible for constitutive clarithromycin resistance (21, 61).

All liquid bacterial cultures were grown in standard mycobacterium medium, Middlebrook 7H9 broth (BD Difco) supplemented with 0.5% albumin, 0.2% glucose, 0.085% sodium chloride, 0.0003% catalase, 0.2% glycerol, and 0.05% Tween 80. Solid cultures were grown on Middlebrook 7H10 agar (BD Difco) supplemented with 0.5% albumin, 0.2% glucose, 0.085% sodium chloride, 0.5% glycerol, 0.0003% catalase, and 0.006% oleic acid.

### Single-point growth inhibition screening assay.

The drug library and collection of in-house antibiotics was screened in microtiter plates as previously described (62) with minor modifications. Briefly, the screen was carried out in 96-well flat-bottom Corning Costar cell culture plates at a single-point concentration of 20 μM with a starting inoculum of an optical density at 600 nm (OD_600_) of 0.05 (10^7^ CFU/ml) in a final volume of 200 μl. The culture for the starting inoculum was diluted from a preculture at mid-log phase (OD_600_, 0.4 to 0.6). The plates were sealed using a Breathe-Easy sealing membrane (Sigma-Aldrich), put in an airtight container with moist tissue, and incubated for 3 days at 37°C on an orbital shaker at 110 rpm. Each plate had a medium-only control and a drug-free control, as well as a positive control, clarithromycin at 20 μM. After 3 days of incubation, the cultures in the wells were manually resuspended before the OD_600_ was read in a TECAN Infinite Pro 200 plate reader. Compounds were defined as hits if they showed growth inhibition of 80% or more of the treated culture compared to the untreated culture. The experiment was conducted in duplicate, and the results are shown as a scatter plot, with each data point representing the mean of data from the two replicates for each compound (Fig. 1).

### Growth inhibition dose-response and bactericidal assays.

MICs in dose-response assays were determined by the broth microdilution method as described previously (63), with some modifications. Briefly, 96-well plates were filled with 100 μl of 7H9 medium in each well. Two times the desired highest final concentration of compound was added to the first well in each row. A 10-point 2-fold serial dilution was carried out. An appropriate dilution of a mid-log-phase culture to an OD_600_ of 0.1 (final OD_600_ in all wells was 0.05) was carried out, and 100 μl of the bacterial culture was added to the wells. The plates were incubated at 37°C and 110 rpm on an orbital shaker for 3 days and then manually resuspended, and the OD_600_ was measured using the plate reader. We report MIC_90_s, which is the concentration that inhibits 90% of growth compared to the untreated control and corresponds to the standard “no visible growth” MIC. In one growth inhibition experiment (Table 4), cation-adjusted Mueller-Hinton broth (36, 37) was used instead of Middlebrook 7H9. All other parameters were kept constant.

**TABLE 4:** Potencies of rifamycins and clarithromycin against reference strains representing subspecies of the M. abscessus complex in Middlebrook 7H9 broth versus cation-adjusted Mueller Hinton broth^*a*^

Strain (*erm*[41] sequevar)^*b*^	MIC_90_ in 7H9 (μM)^*c*^				MIC_90_ in CAMH (μM)			
	RFB	CLR	RIF	RFP	RFB	CLR	RIF	RFP
M. abscessus subsp. abscessus ATCC 19977 (T28)	3	3	37	31	6	0.7	200	84
M. abscessus subsp. bolletii CCUG 50184-T (T28)	4	5	>50	31	9	1	>200	100
M. abscessus subsp. massiliense CCUG 48898-T (deletion)	1	0.4	39	13	3	0.3	>200	50

a The experiments were repeated independently two times, and mean values from those experiments are shown. Standard deviations were ±50% of the values shown. The rifabutin results shown are from drug purchased from Sigma-Aldrich. The MIC experiments were repeated with drug purchased from a different source, Adooq BioScience, and delivered identical results. 7H9, Middlebrook 7H9; CAMH, cation-adjusted Mueller Hinton broth.

b Clarithromycin resistance gene *erm*(41) sequevars: T28 indicates inducible clarithromycin resistance; deletion of *erm*(41) indicates a nonfunctional gene and thus a clarithromycin-sensitive strain.

c RFB, rifabutin; CLR, clarithromycin; RIF, rifampin; RFP, rifapentine.

For bactericidal activity determinations, cultures were grown as described for the MIC determinations with the difference that the cultures were plated on agar for CFU enumeration at the end of the experiment. After 3 days of drug exposure, wells were manually resuspended and 10 μl of the cultures from the first clear well onward were plated at different dilutions on 7H10 agar. The plates were incubated at 37°C for 4 days, and then colonies were counted. We report the MBC_90_, which is the concentration of drug that results in a 90% reduction in CFU/ml of the treated culture compared to the untreated control at time zero.

### Rifabutin preexposure assay.

To determine whether preexposure of M. abscessus to subinhibitory concentrations of rifabutin triggers induction of any (unknown) rifabutin resistance mechanisms, rifabutin preexposure experiments were carried out. A mid-log-phase culture was diluted to an OD_600_ of 0.05 and treated with rifabutin at a subinhibitory concentration of 0.5 μM for M. abscessus subsp. abscessus and M. abscessus subsp. bolletii and 0.1 μM for M. abscessus subsp. massiliense, 4-fold lower than their MIC_50_s (concentrations that causes 50% growth inhibition). An untreated culture was set up as a control. Cultures were grown to mid-log phase overnight and then subjected to standard dose-response determination as described above.
